# A Certificateless Aggregate Arbitrated Signature Scheme for IoT Environments

**DOI:** 10.3390/s20143983

**Published:** 2020-07-17

**Authors:** Dae-Hwi Lee, Kangbin Yim, Im-Yeong Lee

**Affiliations:** 1 Department of Computer Science and Engineering, Soonchunhyang University, Asan 31538, Korea; leedh527@sch.ac.kr; 2 Department of Information Security Engineering, Soonchunhyang University, Asan 31538, Korea; yim@sch.ac.kr

**Keywords:** IoT, certificateless signature, aggregate signature, arbitrated signature, public key replace attack

## Abstract

The Internet of Things (IoT) environment consists of numerous devices. In general, IoT devices communicate with each other to exchange data, or connect to the Internet through a gateway to provide IoT services. Most IoT devices participating in the IoT service are lightweight devices, in which the existing cryptographic algorithm cannot be applied to provide security, so a more lightweight security algorithm must be applied. Cryptographic technologies to lighten and provide efficiency for IoT environments are currently being studied a lot. In particular, it is necessary to provide efficiency for computation at a gateway, a point where many devices are connected. Additionally, as many devices are connected, data authentication and integrity should be fully considered at the same time, and thus digital signature schemes have been proposed. Among the recently studied signature algorithms, the certificateless signature (CLS) based on certificateless public key cryptography (CL-PKC) provides efficiency compared to existing public key-based signatures. However, in CLS, security threats, such as public key replacement attacks and signature forgery by the malicious key generation center (KGC), may occur. In this paper, we propose a new signature scheme using CL-PKC in generating and verifying the signature of a message in an IoT environment. The proposed scheme is a certificateless aggregate arbitrated signature, and the gateway aggregates the signatures of messages generated by the device group to reduce the size of the entire signature. In addition, it is designed to be safe from security threats by solving the problems caused by public key replacement attacks and malicious KGC, and adding arbitrated signatures of the gateway to strengthen non-repudiation.

## 1. Introduction

The Internet of things (IoT) means an environment or technology in which heterogeneous devices are connected to the Internet. The devices participating in the IoT environment can be connected to the Internet to provide various services to users. Thus, the services provided to users through such environments and technologies can also be called IoT. Servers process data collected by “things” (end devices), such as sensors and actuators, and users are provided with services through their smartphones. The most common IoT service structures are things and gateways, storage or servers, and consumers. It consists of things that collect data or perform commands, a gateway that is an intermediate that transmits data collected from things, a storage or server that stores and analyzes data in the form of data desired by users, and consumers who use it. Recently, with the advent of an IoT-based hyper-connected society, technological innovation is ongoing in various fields. In particular, as the fifth-genereation (5G) telecommunications standard has recently attracted attention, the number of IoT devices connected to the Internet will increase rapidly, and various services can be provided [1,2,3,4].

With the development of communication technology, weight reduction and mass production of devices became possible. Since then, following smart homes, more devices are evolving into large-scale IoT services, such as smart buildings, factories, and cities that connect to the Internet. Objects participating in the IoT service environment are each connected to the Internet to communicate with other objects. The feature of the IoT environment is that all devices need to be connected to the Internet, so small devices, such as electrical outlets and gas valves in smart homes, need to include communication capabilities. Therefore, it is composed of ultra-light and low-power technologies, unlike the existing environment. It is also difficult to apply existing public key infrastructure (PKI) security technologies to IoT devices, so it is necessary to use lightweight cryptographic algorithms that work well in this new environment of ultra-light and low-power devices [5,6].

**Figure 1:**
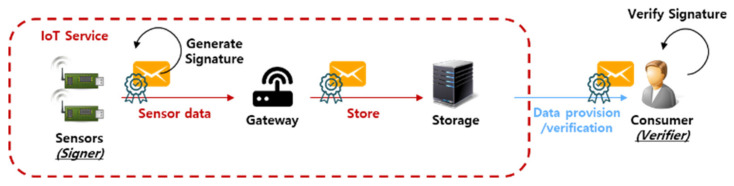
Data signing and verification scenario in an Internet of things (IoT) environment.

In particular, a technology for providing integrity for messages in an IoT environment is essential [7,8]. Figure 1 shows a scenario for the data signing and verification process in an IoT environment. Sensors create a message, create a signature, and place them in cloud-like storage through a gateway. Thereafter, the consumer (i.e., the verifier) who needs the message can secure the integrity of that message through the message and the signature. Since the IoT environment is composed of a wireless communication network, it is possible to provide a secure service only by providing integrity for commands sent by a user to a device or for data collected from a device. Of course, since the IoT is a lightweight environment, the signature must also be lightweight to be applied to that IoT environment. Therefore, in this paper, we analyze the certificateless (CL) signature (CLS) and CL aggregate signature (CL-AS), a signature scheme using the lightweight CL public key cryptography (PKC, hence CL-PKC), which is suitable for IoT environments. In addition, we propose a CL aggregate arbitrated signature (AAS, hence CL-AAS) that applies the gateway arbitrated signature technique to enhance its non-repudiation and the aggregate signature technique to enhance its efficiency. In particular, it is designed to be safe against the forgery of signatures, including against public key replacement attacks that occur in CL-PKC-based cryptographic technologies, and to be suitable for IoT environments by avoiding the use of a large number of computational pairing operations.

The contributions to the proposed scheme in this paper are as follows.

- Analyze existing CL-AS schemes and design scenarios for secure public key replacement and malicious KGC attacks.
- In addition to the existing security requirements, the concept of the arbitrated signature for the non-repudiation function is applied considering the security of the aggregator that aggregates signatures.
- Aggregate signature is performed on messages and signatures of IoT devices, and the arbitrated signature of the gateway is also aggregated in the aggregate signature of IoT devices. Through this, we propose a secure and efficient CL-AAS scheme compared to the existing schemes.

More details on CL-PKC and security threats are covered in Section 2 on related work. Section 3 introduces the security requirements for each item in the cryptosystem, and Section 4 introduces the proposed scheme that satisfies those security requirements. Section 5 gives a comparative analysis of the proposed scheme, with Section 6 giving the paper’s conclusions.

## 2. Background and Related Work

In this section, we consider background and related work, before suggesting the CL-PKC-based CL-AAS scheme to satisfy security requirements and provide efficiency in an IoT environment. Even before wireless sensor networks had become common, research had been conducted on digital signatures to provide message integrity. Some cryptography schemes have been used recently in IoT environments, such as the CLS and CL-AS used in the signature process proposed in this paper; we now examine these, including their security threats.

### 2.1. Elliptic Curve Cryptography and ECDLP

The elliptic curve cryptography (ECC) is a public key cryptography based on the elliptic curve theory, and provides a similar level of security while using a shorter key than the existing public key cryptography. Therefore, it is applied to various cryptographic algorithms used in IoT environments. The definition of the elliptic curve cipher is as follows.

Let $F_{q}$ denote a finite field with a large prime order q. Let $E_{q}$ denote an elliptic curve on $F_{q}$, which is specified by the equation: $y^{2}=x^{3}+ax+b\left(mod\;p\right)$, where $a,b\in F_{q}$ and $\left(4a^{3}+27b^{2}\right)mod\;p\neq 0$. Let the notation *O* denote a point of infinity, form the additive cyclic group *G* of the elliptic curve under the computation of point addition T=U+V for U,V∈G defined on the basis of a chord-and-tangent rule. Suppose *P* is a generator of the cyclic group *G*, and the order of *G* is q. Let $x\in Z_{q}^{*}$, and scalar multiplication is defined by the equation: $x\times P=\left(P+P+\ldots +P\right)$ (x times).

The ECC was designed based on the elliptic curve discrete logarithm problem (ECDLP). Given two random points P,Q∈G, the ECDLP is to find the integer $x\in Z_{q}^{*}$, where Q=x×P.

### 2.2. Digital Signature

Digital signature is a security technology in which a signature has been changed into a digital form; it serves as proof of the identity of a digital electronic document’s author. Digital signature is a cryptographic technology created by PKI-based public key cryptography (PKC) technology, starting with the authentication of messages [9,10,11]. Authentication can be divided into that of the user and that of the message. The former confirms the identity of a valid user and is a basic element for ensuring the responsibility of users. The latter may, for instance, provide assurance that the received message has not been tampered with. Digital signature consists of a signature using a private key, a verification using a public key, and provides a non-repudiation function, to ensure that it was sent from the sender. For digital signatures, the following five items must be satisfied:

- Signer authentication: The signer of the electronic document must be verifiable;
- Unforgeable: The electronic document cannot be forged;
- Non-reusable: The electronic signature cannot be used as a signature for another document;
- Unmodifiable: The content of the electronic document cannot be changed; and
- Non-repudiation: The signature of the electronic document cannot be denied.

Digital signatures began with PKC and various other types have been developed, including blind, arbitrated, group, multi-, and one-time signatures, and a variety of AS schemes that aggregate multiple signatures into one [9,11,12,13,14,15,16,17,18].

### 2.3. Certificateless PKC (CL-PKC)

There is a problem with PKC in that it is difficult to apply in an environment requiring ultra-light and low-power devices, such as the IoT. In particular, PKI, upon which PKC is based, uses a certificate, to verify the public key for authentication of the user, and the user’s public key. For this reason, the computational overhead is exceptionally large for managing keys, signatures, and certificates and their distribution, verification, and revocation.

To solve these problems of PKC, identity-based cryptography (IBC) was developed [19]. Since IBC uses a public key based on a known user’s identifier, it can solve the problems of key distribution, certificate verification, and memory overhead by eliminating the public key verification process. However, identity-based encryption has a problem with key escrow [20,21]. In IBC, there is a key generation center (KGC) that receives a user’s identifier to generate a private key based on it and returns this to the user. The direct generation of the user’s private key in this way can expose the user’s key to the KGC afterwards: The key escrow problem.

One proposed scheme to solve the key escrow problem in IBC is CL-PKC [22], in which the KGC does not generate all public and private keys but only partially generates them and returns them to the user. This “partial” key is called a partial secret key (PSK), and the user creates his or her full key pair using this. Since the full key pair is not generated by the KGC but by the user, CL-PKC can solve the key escrow problem, uses a smaller key than the existing PKI, and does not incur the overhead for public key verification; it is thus suitable for ultra-light and low-power environments. Research has been conducted on various aspects of CL-PKC, such as authentication and key agreement [23,24], signatures [25,26], and encryption [27,28]. The main difference between the structures of CL-PKC and its predecessor, PKC, is that there is no certificate for public key verification in the former and, in this sense, the term “certificateless” is used.

### 2.4. Certificateless Aggregate Signature (CL-AS)

Before explaining CL-AS, we describe CLS, which is the basis for it. CLS is a signature technique using CL-PKC: The signature for a message is generated using the private key of the signer, generated via CL-PKC, and the verifier verifies it using the public key and identifier of the signer and the public key of the KGC. CLS and CL-PKC are currently active research topics [22,25,26].

**Figure 2:**
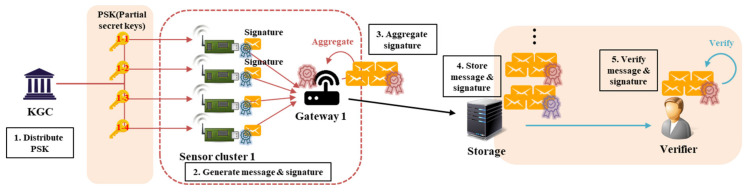
Structure of the general certificateless aggregate signature process. KGC: key generation center.

CLS is a scheme for signing a single message, whereas CL-AS is a scheme for creating an aggregated signature for multiple messages. If there are multiple senders, the signature on the generated messages will generate the signature of each sender. That is, N signatures are generated for N messages generated by N senders. The verifier must perform verification of N messages individually, using N public keys of N senders. Thus, the number of individual verifications will, likewise, be N. CL-AS aggregates these N signatures into one signature. N public keys are used for verification, but the advantage of being able to verify all of the signatures for N messages in one step is that only one verification process is required. This can reduce the computational overhead on the verifier side and, on the storage side (messages and signatures must be stored), only one signature, not N of them, needs to be stored, thereby reducing memory overhead. The CL-AS scheme was proposed based on a pairing operation, but, recently, pairing-free schemes to reduce the number of operations have been proposed [29,30,31,32,33,34]. Figure 2 shows the structure of CL-AS.

The basic CL-AS schemes include a CL-PKC-based signature that receives a PSK after registering a user with a KGC. In general, the CL-based AS technique consists of the following eight steps. Among them, the setup and partial-private-key-extract steps are performed by the KGC, and the set-secret-value and set-public-key steps are performed by the user who generates the key. Thereafter, the signer who wants to generate the signature does so for the message with his key through the CL-sign step and can verify the message and signature through the CL-verify step. Signatures generated from multiple signers are sent to the aggregator through the CL-aggregate step, which aggregates them into one signature, and then on to the verifier. The verifier can acquire and verify messages through the CL-aggregate-verify step.

- Setup: The KGC generates public parameters and a master secret key using a security parameter as input.
- Partial-private-key-extract: The KGC generates the user’s partial private key and partial public key using the public parameters, master secret key, and the user’s personal identification information, and delivers these keys to the user.
- Set-secret-value: The user creates his own secret information and secret key by inputting public parameters and user identification information.
- Set-public-key: The user sets the public key by entering the public parameters, his partial public key, and secret information.
- CL-sign: Among the users who generated the key, the user who wants to sign the message becomes a signer, and signs the message using his private key. The message and its signature are sent to the verifier.
- CL-verify: The verifier verifies the integrity of individual messages and signatures using the signer’s public key.
- CL-aggregate: The aggregator, which receives the messages and signatures from multiple signers, aggregates these signatures into a single one for multiple messages, to reduce their overall size, and outputs this.
- CL-aggregate-verify: Upon receiving a message and an aggregated signature, the verifier can verify the signature using the signer’s public key, verify the user who created the signature, and verify the integrity of the message.

### 2.5. Security Threat of CL-AS

CL-AS has several advantages, but it also has problems with the forgery of messages and signatures. The public key used in CL-PKC does not use a certificate, so the user’s identifier and public key cannot be authenticated. Because of this, CLS has a weaker non-repudiation function than PKI, and a malicious attacker can conduct an attack in which another user’s public key is replaced. This CLS public key replacement attack is a method of forging the signature transmitted by device A to device B and replacing the public key of user A with a public key generated by the attacker to verify that forged signature. This is an attack that occurs because it is not possible to authenticate whether the public key, which can bypass the verification of the signature generated using A’s private key, is that of A or that of the attacker. It can be verified that the signature using the public key of device A, but the function of non-repudiation to verify the signature is actually signed by A is weak. The act of being able to verify by replacing the public key itself is an infringement of non-repudiation, and in order to prevent this, CL-PKC must be considered for a public key replacement attack. Additionally, unlike IBC, there is no problem of key escrow for the device’s secret key; however another problem may also occur: The KGC can generate the partial key of A and forge user A’s signature using a partial key generated by itself.

Therefore, the security model for CL-AS can be roughly divided into two types of attack model [29,30,31,32,33,34]. Each model is of a game by an attacker ($A_{I}$ or $A_{II}$) communicating with a challenger to successfully forge a signature. $A_{I}$ has the ability to arbitrarily replace the public key of a legitimate user without the system’s master key. $A_{II}$ cannot replace the public key of users but knows the master secret key of the KGC. Thus, each of them can perform different types of attacks.

### 2.6. Analysis of Existing CL-AS Schemes

CLS was first proposed by Al-Riyami et al. in 2003 [21]. Based on this, the recently proposed CL-AS has had many implementations published that are more efficient because they do not use pairing operations. Table 1 summarizes some of these variants and their security threats. 

Qu et al. [29] proposed an efficient CL-AS scheme that does not use a pairing operation. Most commonly, CL-AS structures are based on the elliptic curve discrete logarithm problem (ECDLP). This scheme is one such and adds the user-generated key and PSK to the signature. However, since the identifier is not bound to the public key, there is a risk that the public key can be replaced.

Deng el al. [30] proposed a CL-AS scheme that prevents forgery of signatures by adding two kinds of signatures in one signature statement, by adding an RSA signature along with an ECDLP-based Schnorr signature. However, the size of the signature statement for the message is exceptionally large, and the signature verification overhead is disadvantageous because two types of signatures must be verified. In particular, the former signature is based on an exponential operation, unlike the latter signature, which uses an elliptic curve, so it has a large overhead compared to other schemes.

Cui et al. [31] proposed a scheme to prevent the transmission of a forged signature due to the replacement of the public key by adding a timestamp when sending the signature and message. However, since the identifier is not actually bound to the public key, it does not provide direct defense against a public key replacement attack.

Du et al. [32] proposed a scheme that was safe against public key replacement attacks by binding an identifier and a verification key to a public key, but there is a risk of key leakage and subsequent signature forgery.

Gayathri et al. [33] proposed a scheme to aggregate public parameters for signature verification. Previous schemes required N public parameters to verify N signatures, but Gayathri et al. could reduce the memory overhead by using a scheme that reduces the verification parameters. However, there is a risk of a public key replacement attack because the identifier is not bound to the public key.

Zhao et al. [34] proposed a scheme to prevent signature forgery by adding a value for verifying the signature directly to the overall transmitted value of the message and the aggregated signature. However, the message that is transmitted is exceptionally large, and there is still a risk of a public key replacement attack.

The above schemes suggest CL-AS for various environments. The security analysis and efficiency comparison of existing schemes are summarized in Section 5 and Section 6. In general, the risk of public key replacement attack occurs when the user, identifier, and verifiable value are not bound to the PSK received via the KGC. In other words, the signature can be verified with the public key of the device A, but it occurs when the public key used to verify the signature cannot verify whether the public key generated by the object with the actual identifier A is correct. This means that the existing problem is related to non-repudiation, and this problem can be solved if the public key can verify the identity of the identifier A. To solve this, in recent CL-PKC schemes, the partial key is received from the KGC first, and the user does not generate the full key but, instead, first generates the verification key pair and sends the identifier and public key for verification to the KGC. Using this, the KGC binds the user’s identifier, the public key, for verification, and the verification tag to the PSK, and then enables verification of the user’s public key [35,36]. Additionally, in the existing CL-AS schemes, the aggregator that aggregates the signatures of the signer’s messages serves solely to aggregate these signatures. The aggregator can be one of the signers or a third entity, depending on the environment. If it is a third entity, the problem of trusting it may occur, which can make the AS unreliable. Therefore, it is necessary that the aggregator in CL-AS has non-repudiation.

## 3. Security Requirements

- Integrity: The most important requirement for digital signatures, including CLSs, is integrity. In particular, in the IoT environment, since data are transmitted and received using a wireless communication network, it is particularly important to ensure integrity by signing important messages. In the existing CL-AS schemes, since the aggregator only aggregates the signature, the entity that verifies the signer’s signature first is that aggregator. The integrity of the aggregate signature itself must be ensured, as it can also be an attack point.
- Prevention of key leakage: The reason for performing the signature is to ensure the integrity of the transmitted message, and the signer’s signature key must not be leaked to the outside or be possible to derive via public parameters. If an attacker can derive or steal the signature key, they can forge the signature on messages generated by themselves, reducing the reliability of the IoT service, and create and transmit a malicious message that the attacker can have verified legitimately.
- Unforgeability: An attack on CL-PKC-based signatures is an attack with counterfeit signatures. As described in Section 2.4, forgery of signatures can occur through the public key replacement attack of adversary $A_{I}$ or the generation of the signer’s partial key using the KGC master key of adversary $A_{II}$. For adversary $A_{I}$, even if public key replacement is performed, it should not be possible to generate a valid signature. If the verifier could remove the private key portion of the signature using the replaced public key, the attack would succeed. In particular, since a public key certificate is not used in CL-PKC-based cryptographic protocols, it is essential to verify that the public key used for signature verification is the actual signer’s public key, and the user’s identifier and public key cannot be authenticated. So, the non-repudiation function must be strengthened. For adversary $A_{II}$, it should not be possible to generate a signature using only the signer’s partial key. This means that both the PSK and the signer-generated key must be used when generating the signature. In addition, even if the signature is generated using both, the signature can be forged, so the verifier should not be able to verify the forged signature normally.

## 4. Proposed Scheme

**Figure 3:**
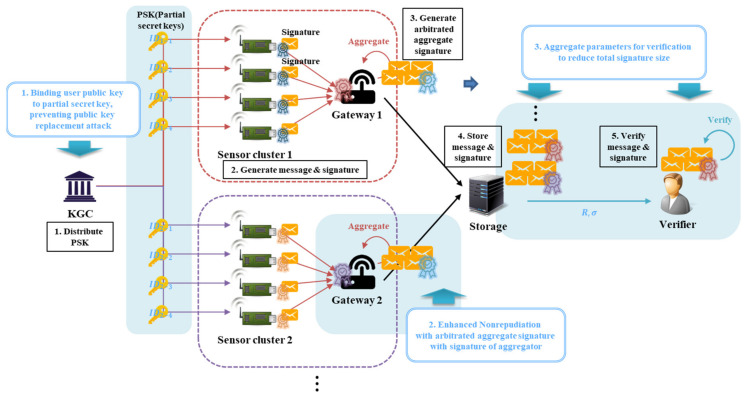
Scenario and advantages of the proposed scheme. KGC: key generation center.

In this paper, we propose CL-AAS, a scheme with an aggregated and arbitrated signature, for IoT environments. Figure 3 shows the proposed scenario. In the IoT environment, sensor devices act as signers to generate messages and directly generate signatures. Sensor devices gather to form a sensor cluster, and each cluster has a gateway. A message is generated from the sensor device, and each device signs the message through the private key generated by CL-PKC and sends it to the gateway, which simultaneously acts as an arbitrator and an aggregator.

As a feature of the proposed scheme, it is possible to strengthen the non-repudiation of the signature of the sensor device through the arbitrated signature of the gateway, and reduce both the size of the signature stored in storage and the verification overhead of the verifier through the AS. In the existing CLS scheme [37,38], an entity, such as an external time server or “helper”, synchronizes with the signer to strengthen non-repudiation for the signature. In the IoT scenario proposed in this paper, the messages generated by the sensor device are aggregated and transmitted through the gateway, so we do not use other external entities but try to strengthen the non-repudiation by using the gateway itself. In other words, the gateway does not merely act as an aggregator for combining multiple signatures into a single one but also makes it an arbitrated signature.

The system parameters of the proposed scheme are as follows.

- $ID_{*}$: Identifier of entity;
- E: Elliptic curve on group *G* of prime order *q*;
- P: Generator of cyclic group *G*;
- $pu_{*},sv_{*}$: Verification the public key and private key pair of entity;
- $PU_{*},PR_{*}$: Full public key and private key pair of entity;
- msk: Master key of KGC;
- $P_{Pub}$: Public key of KGC $\left(P_{Pub}=msk\times P\right)$;
- $D_{*}=\left(R_{*},z_{*}\right)$: Partial key of the entity;
- $H_{1}\left(\cdot \right)$: Cryptographic hash function $\left({\left\{0,1\right\}}^{*}\times G\times G\to Z_{q}^{*}\right)$;
- $H_{2}\left(\cdot \right)$: Cryptographic hash function $\left({\left\{0,1\right\}}^{*}\times {\left\{0,1\right\}}^{*}\times G\times G\to Z_{q}^{*}\right)$; and
- $H_{3}\left(\cdot \right)$: Cryptographic hash function $\left({\left\{0,1\right\}}^{*}\times {\left\{0,1\right\}}^{*}\times Z_{q}^{*}\times G\times G\to Z_{q}^{*}\right)$.

The proposed CL-AAS scheme consists of four phases: Setup, individual signing and verifying, aggregated arbitrated signing, and aggregated verifying. In the setup phase, the KGC sets the public parameters and distributes the participants’ partial keys. In the individual signing and verifying phase, the participants (such as devices) use their partial keys to generate individual signatures and the aggregator verifies them. In the aggregated arbitrated signing phase, the messages and signatures of all the signer devices are turned into one signature and the gateway adds its arbitrated signature.

Finally, in the aggregate verify phase, the signatures of the devices and the signature of the gateway’s arbitrated signature are verified at once.

Each phase of the proposed scheme is modified from the eight algorithms described in Section 2.3. The set-secret-value and set-public-key algorithms are replaced with set-device-key and set-full-key ones, respectively. This is because the verification key pair of the user is generated first, not the partial key. Additionally, the AS (aggregated signature) and verification algorithms are replaced by CL-AA-sign to generate the AAS (aggregate arbitrated signature) and CL-AA-verify for aggregate verification. Therefore, this proposed scheme consists of the following eight algorithms for the KGC; A; gateway, G, acting as an aggregator and arbitrator; and verifier, V:

- Setup $\left(k\right)$: The KGC creates public parameters and a master secret key with a security parameter, k, as input.
- Set-device-key $\left(params,ID_{A}\right)$: A generates a verification key pair from the public parameters, params, and A’s public identifier, $ID_{A}$.
- Partial-private-key-extract $\left(params,msk,ID_{S},pu_{S}\right)$: The KGC uses params, the master secret key, *msk*, $ID_{A}$, and the verification public key, $pu_{A}$, to generate the partial key, $D_{A}$, of A and transmits it to A.
- Set-full-key $\left(params,ID_{A},D_{A},pu_{A},sv_{A}\right)$: A sets its full key pair, $PU_{A},PR_{A}$, using params, $D_{A}$ received from the KGC, and the verification key pair, $sv_{A},pu_{A}$.
- CL-sign $\left(m_{A},ID_{A},PR_{A},PU_{A}\right)$: A becomes a signer, and signs a single message, $m_{A}$, using its private key, $PR_{A}$. Then, $m_{A}$ and its signature are transmitted to G.
- CL-verify $\left(m_{A},\sigma_{A},ID_{A},PU_{A}\right)$: Verification of $m_{A}$ and its signature, $\sigma_{A}$, is performed using $ID_{A}$ and the public key, $PU_{A}$. In the proposed scheme, the gateway performs verification, and the signatures of all received messages are verified through this process.
- CL-AA-sign $\left(m_{1},\ldots ,m_{n},\sigma_{1},\ldots ,\sigma_{n},ID_{1},\ldots ,ID_{n},ID_{G},PU_{1},\ldots ,PU_{n}\right)$: G, which has received messages and signatures from multiple devices, reduces the size of the signature. The signature is aggregated through the process, and an arbitrated signature is added: This algorithm outputs one signature that has been aggregated for multiple messages. To reiterate, G creates a single aggregated signature for all the signatures of the devices.
- CL-AA-verify $\left(m_{1},\ldots ,m_{n},\sigma_{AS},ID_{1},\ldots ,ID_{n},ID_{G},PU_{1},\ldots ,PU_{n}\right)$: When V receives the message and its aggregated signature from G, the signature and public keys can be used to verify the signature and, thus, the integrity of the message.

**Figure 4:**
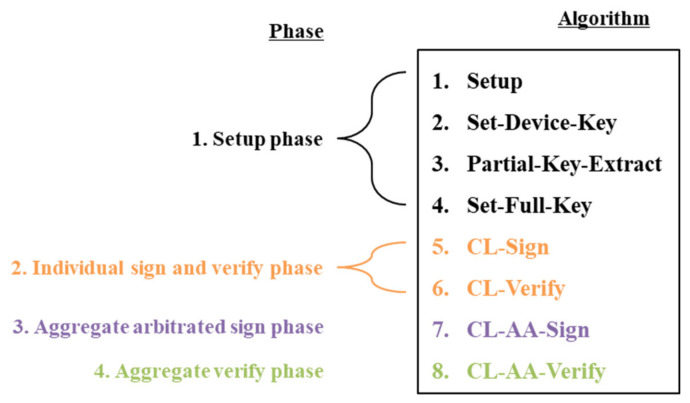
The relationship between the phases and algorithms of the proposed scheme. CL: certificateless, AA: aggregate arbitrated.

Figure 4 shows the four phases and eight algorithms of the proposed scheme.

### 4.1. Setup Phase

**Figure 5:**
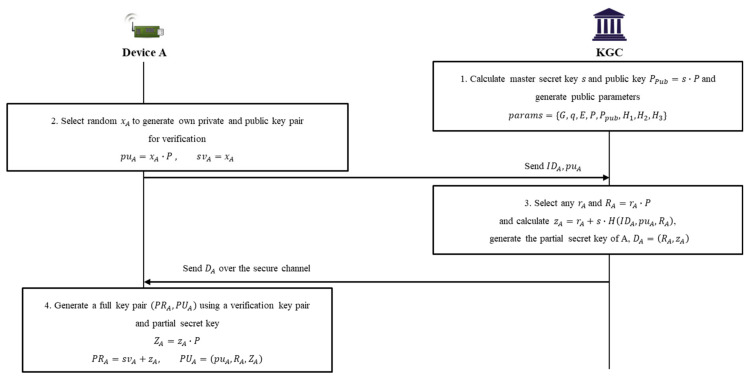
Sequence diagram of setup phase.

In the setup phase, KGC first performs the setup algorithm using *k* (the security parameter) to generate the initial parameters, *msk* (the master secret key), and a master public key, $P_{Pub}$. The KGC is responsible for generating the partial keys of the devices after generating *params* (the public parameters). Subsequently, a verification key pair for each device is generated through the set-device-key algorithm; then, a partial key is generated for each by performing the partial-private-key-extract algorithm by sending information to the KGC. A device that receives a PSK generates its own full key pair through the set-full-key algorithm. Figure 5 shows the sequence diagram of the setup phase.

Step 1. The KGC selects k and generates *msk*. After that, $P_{pub}=msk\times P$ and params are generated as follows, through the setup(k) algorithm:

(1) $$params=\left\{G,q,E,P,P_{pub},H_{1},H_{2},H_{3}\right\}.$$

Step 2. A, which needs to receive a partial key from KGC, first creates its own verification public and private key pair, $pu_{A},sv_{A}$, through the set-device-key (*params, ID*) algorithm. A selects $x_{A}\in_{R}Z_{q}^{*}$ and computes $pu_{A},sv_{A}$ as follows:

(2) $$pu_{A}=x_{A}\times P,sv_{A}=x_{A}.$$

Step 3. A sends $ID_{A}$ and $pu_{A}$ (its verification public key) to the KGC, which performs the partial-private-key-extract ($params,msk,ID_{A},pu_{A}$) algorithm to generate $D_{A}$ (the partial key). The KGC selects $r_{A}\in_{R}Z_{q}^{*}$ and calculates the result of Equation (3). Then, the result of Equation (4) is calculated to generate a signature for the public key. The KGC transmits $D_{A}=\left(R_{A},z_{A}\right)$ to A over a secure channel:

(3) $$R_{A}=r_{A}\times P,$$

(4) $$z_{A}=r_{A}+msk\times H_{1}\left(ID_{A},pu_{A},R_{A}\right).$$

Step 4. A, which has received $D_{A}$, creates $PR_{A}$ (its full private key) and $PU_{A}$ (its full public key) through set-full-key ($params,ID_{A},D_{A},pu_{A},sv_{A}$) as follows:

(5) $$Z_{A}=z_{A}\times P,$$

(6) $$PR_{A}=sv_{A}+z_{A},\;PU_{A}=\left(pu_{A},R_{A},Z_{A}\right).$$

### 4.2. Individual Signing and Verifying Phase

**Figure 6:**
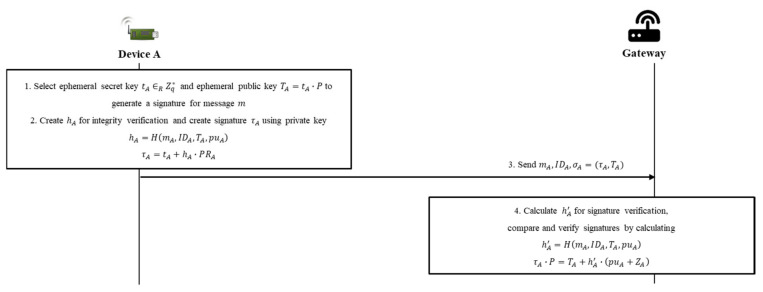
Sequence diagram of individual signing and verifying phase.

The individual signing and verifying phase use the CL-sign and CL-verify algorithms. A, which needs to generate a signature, becomes a signer and signs a message using its own key. Figure 6 shows the sequence diagram of the individual signing and verifying phase.

Step 1. A selects an ephemeral secret key, $t_{A}\in_{R}Z_{q}^{*}$, and calculates an ephemeral public key, $T_{A}=t_{A}\times P$.

Step 2. A needs to send a signed message to the arbitrator, G, to send the message and signature to V, to communicate. A calculates $h_{A}$ and $\tau_{A}$ as follows, to generate the signature for $m_{A}$ (the message):

(7) $$h_{A}=H_{2}\left(m_{A},ID_{A},T_{A},pu_{A}\right),$$

(8) $$\tau_{A}=t_{A}+h_{A}\times PR_{A}.$$

Step 3. A sends $m_{A}$, $\sigma_{A}=\left(\tau_{A},T_{A}\right)$ (the signature for $m_{A}$), and $ID_{A}$ to G.

Step 4. G receives $\sigma_{A},ID_{A},$ and $m_{A}$ and performs the process of verifying $\sigma_{A}$. G calculates $h_{A}^{\prime }$ as in Equation (9), using the information from the message and that has been published by A, and verifies the validity of $\tau_{A}$ via Equation (10). If the validity of $\tau_{A}$ is verified, G completes verification of the individual message and its signature for A. G performs the signature not only for A but also for the messages and signatures of the other devices that will form the aggregate of signatures, as in Equations (9) and (10):

(9) $$h_{A}^{\prime }=H_{2}\left(m_{A},ID_{A},T_{A},pu_{A}\right),$$

(10) $$\tau_{A}\times P=T_{A}+h_{A}^{\prime }\times \left(pu_{A}+Z_{A}\right).$$

The validity of the verified contents can be confirmed as follows:

(11) $$\begin{array}{cc} \tau_{A}\times P & =\left(t_{A}+h_{A}\times PR_{A}\right)\times P\\ & =t_{A}\times P+h_{A}\times PR_{A}\times P\\ & =T_{A}+h_{A}\times \left(pu_{A}+Z_{A}\right). \end{array}$$

### 4.3. Aggregated Arbitrated Signing Phase

**Figure 7:**
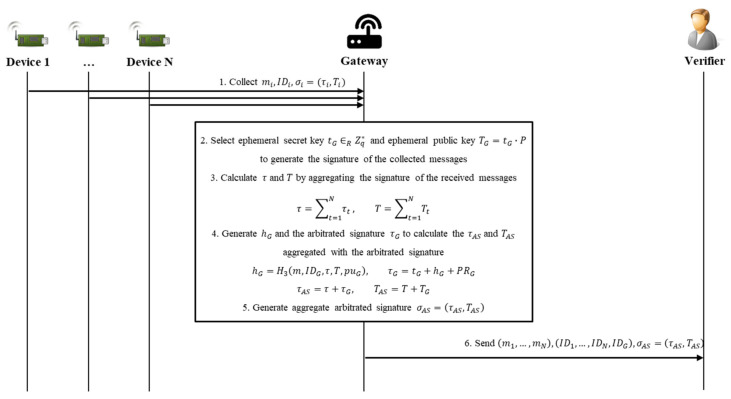
Sequence diagram of aggregated arbitrated signing phase.

In the aggregated arbitrated signing phase, G aggregates signatures on messages and signatures received from N devices, and creates an arbitrated signature that has been verified, and adds it to the aggregated signature. An arbitrated signature is not simply a signature but is also the means of confirming that G has completed verification for the messages and signatures received from each included device; it is generated using G’s own private key. Thus, an aggregated signature is generated, including the devices’ signatures and the gateway-generated arbitrated signature. The aggregated arbitrated signing phase includes the CL-AA-sign algorithm. Figure 7 shows the sequence diagram of the aggregated arbitrated signing phase.

Step 1. Each device sends the content of the message and signature created by itself ($m_{i}$, $ID_{i}$, and $\sigma_{i}$, where these relate to the *i*th device) to G, for which G collected and verified each signature during the signing and verifying phase.

Step 2. G selects an ephemeral secret key, $t_{G}\in_{R}Z_{q}^{*}$, and generates an ephemeral public key, $T_{G}=t_{G}\times P$, to generate the signature of the collected message, $m=\left(m_{1},\ldots ,m_{N}\right)$.

Step 3. G calculates the following to perform aggregation on the actual signature, $\tau_{i}$, constituting the signature, $\sigma_{i}$, and the verification value, $T_{i}$:

(12) $$\tau =\left(\sum_{t=1}^{N}\tau_{t}\right),T=\left(\sum_{t=1}^{N}T_{t}\right).$$

Step 4. G calculates the results of Equations (13) and (14) using its private key, $PR_{G}$, to generate the elements of the arbitrated signature to indicate that it has completed verification of each message. Then, the results of Equation (15) are calculated to generate $\tau_{AS}$ and $T_{AS}$:

(13) $$h_{G}=H_{3}\left(m,ID_{G},\tau ,T,pu_{G}\right),$$

(14) $$\tau_{G}=t_{G}+h_{G}\times PR_{G},$$

(15) $$\tau_{AS}=\tau +\tau_{G},T_{AS}=T+T_{G}.$$

Step 5. Finally, G creates an AAS (aggregate arbitrated signature), $\sigma_{AS}=\left(\tau_{AS},T_{AS}\right)$, that aggregates the arbitrated signature of G and all the signatures of the devices.

Step 6. To verify $\sigma_{AS}$, $\left\{m,\left(ID_{1},\ldots ,ID_{N},ID_{G}\right),\sigma_{AS},\left(PU_{1},\ldots ,PU_{N},PU_{G}\right)\right\}$ must be used. $PU_{i}$ is the published public key of the *i*th device. Therefore, the gateway then sends $\left\{m,\left(ID_{1},\ldots ,ID_{N},ID_{G}\right),\sigma_{AS}\right\}$ to V (the verifier requesting the message).

### 4.4. Aggregated Verifying Phase

**Figure 8:**
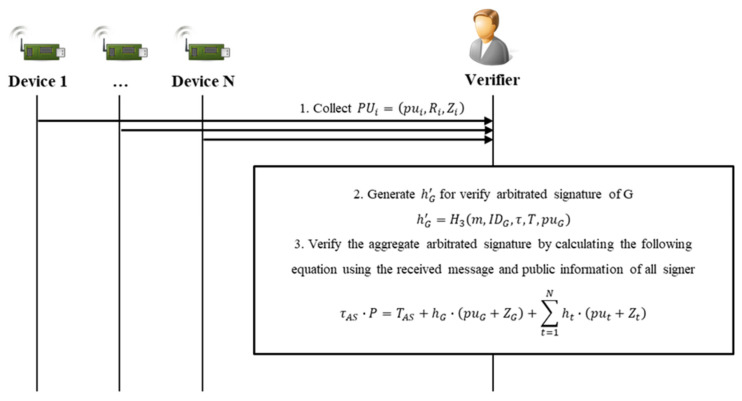
Sequence diagram of aggregated verifying phase.

In the aggregated verifying phase, V verifies the signature and message received from G, and verifies the signer’s signature and G’s arbitrated signature together. During this process, the initial signer and the arbitrator are verified simultaneously, strengthening the non-repudiation function. This involves the final algorithm, CL-AA-verify, of the eight in this scheme. Figure 8 shows the sequence diagram of the aggregated verifying phase.

Step 1. G can store the generated $\sigma_{AS}$ and the message in a repository or send it directly to V. V performing the verification receives $\left\{m,\left(ID_{1},\ldots ,ID_{N},ID_{G}\right),\sigma_{AS}\right\}$ from G and confirms the identifiers of the devices to obtain the public keys, $PU_{i}=\left(pu_{i},R_{i},Z_{i}\right)$.

Step 2. V calculates the value $h_{G}^{\prime }$ for the arbitrated signature verification of G as follows:

(16) $$h_{G}^{\prime }=H_{3}\left(m,ID_{G},\tau ,T,pu_{G}\right).$$

Step 3. V can verify the validity of $\sigma_{AS}$ by calculating the result of Equation (17). If valid, V has completed verification of the N signer devices and G’s arbitrated signature in one step:

(17) $$\tau_{AS}\times P=T_{AS}+h_{G}\times \left(pu_{G}+Z_{G}\right)+\sum_{t=1}^{N}h_{t}\times \left(pu_{t}+Z_{t}\right).$$

The validity of the verified contents can be confirmed as follows:

(18) $$\begin{array}{cc} \tau_{AS}\times P & =\left(\tau_{1}\times P+\ldots \tau_{N}\times P+\tau_{G}\times P\right)\\ & =\left(t_{1}+h_{1}\times PR_{1}\right)\times P+\ldots +\left(t_{N}+h_{N}\times PR_{N}\right)\times P+\left(t_{G}+h_{G}\times PR_{G}\right)\times P\\ & =\left(T_{1}+h_{1}\times \left(pu_{1}+Z_{1}\right)\right)+\ldots +(T_{N}+h_{N}\times \left(pu_{N}+Z_{N}\right)+(T_{G}+h_{G}\times \left(pu_{G}+Z_{G}\right)\\ & =\left(T_{1}+\ldots +T_{N}+T_{G}\right)+\left(h_{1}\times \left(pu_{1}+Z_{1}\right)\right)+\ldots +\left(h_{N}\times \left(pu_{N}+Z_{N}\right)\right)+\left(h_{G}\times \left(pu_{G}+Z_{G}\right)\right)\\ & =T_{AS}+h_{G}\times \left(pu_{G}+Z_{G}\right)+\sum_{t=1}^{N}h_{t}\times \left(pu_{t}+Z_{t}\right). \end{array}$$

## 5. Security Analysis

This section describes how the proposed scheme satisfies the security requirements presented in Section 3, including the requirements regarding integrity, key leakage, and forgery.

### 5.1. Integrity

In CLS or CL-AS, an existing CL-PKC-based signature scheme, a Schnorr signature, is used. In the proposed scheme, the individual signature value, $\tau_{A}$ in $\sigma_{A}$, of A is in the same form as the Schnorr signature, and the tag for verifying this is $T_{A}$. $\tau_{A}$ is created using A’s private key, $PR_{A}$, and the tag’s secret value (its ephemeral secret key), $t_{A}$, and can be verified through $PU_{A}$. Here, the value that is actually verified is the content of the hashed value, $h_{A}$. The values hashed from $h_{A}$ are the message, $m_{A}$; the identifier, $ID_{A}$, of the device, A, that was the signer; the verification public key, $pu_{A}$; and the verification tag, $T_{A}$. Eventually, $h_{A}$ is signed using $PR_{A}$ to provide the integrity of the message and can be verified using $pu_{A}$ and the partial public key, $Z_{A}$, which are elements of $PU_{A}$ for verification. Therefore, it indicates that the signature, $\sigma_{A}$, was generated by A and was not forged in “the middle”, between A and V.

In addition, the aggregated signature, $\sigma_{AS}$, generated by the gateway, G, using the individual signatures is added to the $\tau_{i}$ and $T_{i}$ of the signers who generated the message, and to $\tau_{G}$ and $T_{G}$, the arbitrated signature values generated by G with the private key, $PR_{G}$. G’s signature takes the form of a Schnorr signature, just like for the messages generated by the other devices, and can be verified in the same way. However, there is one difference: The content of the arbitrated signature being verified, $h_{G}$, is the message set, m, and the identifier, $ID_{G}$, of G and the aggregated devices’ signature elements, τ and T. G can ultimately provide the integrity by signing the signature set itself of messages received from devices via $\sigma_{AS}$, and can be verified using the public key, $PU_{G}$, of G. If the message of the device is forged, the verification of the individual signature or AAS will fail, and only the normal message can be verified.

### 5.2. Prevention of Key Leakage

The signing key (full private key) and verification key (full public key) used in the proposed scheme are CL-PKC-based key pairs generated by the KGC and the device itself. It is assumed that when a device is first issued a partial key by the KGC, this is transmitted through a secure channel. In addition, all other messages and signatures that are transmitted are transmitted through a public channel. In the individual signing and verifying phase, the message sent to the public channel is the entire message, $m_{A}$, $ID_{A}$, and $\sigma_{A}$, and if they can derive the signing key, the attacker will succeed in leaking the key. In the aggregated arbitrated signing phase, the entire message transmitted to the public channel is $m,\left(ID_{1},\ldots ,ID_{N},ID_{G}\right),$ and $\sigma_{AS}$, and if $\sigma_{AS}$ can derive the signing key, the key can be successfully leaked.

First, $\sigma_{A}$ is composed of $\tau_{A}=t_{A}+h_{A}\times PR_{A}$ and $T_{A}=t_{A}\times P$. The public key, $PU_{A}$, of A consists of $pu_{A}$, $R_{A}$, and $Z_{A}$, the values used in the verification of $\sigma_{A}$ are $pu_{A}$ and $Z_{A}$, and $R_{A}$ is used to verify the validity of the PSK, $z_{A}$, generated by the KGC. Furthermore, it can be verified that the public key, $Z_{A}$, was made by A and the KGC. 

The signature is verified as in Equation (10) and, even if the attacker knows the public key and other published information, obtaining the signature key from the signature is the same as the difficulty of solving the ECDLP in $\left(pu_{A}+Z_{A}\right)=\left(sv_{A}+z_{A}\right)\times P$. Therefore, it is difficult for an attacker to derive a public key using disclosed information. In particular, in the proposed method, since each value in the form of the signature key, $\left(sv_{A}+z_{A}\right)$, is used as a single value by adding them together, rather than independently using $sv_{A}$ and $z_{A}$ as in many existing schemes, this helps against leaking keys: There are fewer threats. Similarly, in $\sigma_{AS}$, calculating the private keys of each signature using $\tau_{AS}$ and $T_{AS}$ is the same as solving the ECDLP problem, so it is difficult to leak or derive the signature key from the proposed scheme.

### 5.3. Unforgeability

As described in Section 3, the attack that can occur in a CL-PKC-based signature protocol is, in fact, an attack on unforgeability. A signature protocol is insecure if a tampered signature on any message can be verified (as if it were legitimate). Attacks on unforgeability can be divided into those by $A_{I}$ and those by $A_{II}$.

#### 5.3.1. Unforgeability from Adversary $A_{I}$

The adversary $A_{I}$ has the ability to replace the public keys of other users with one generated by themselves. Due to the safety of the ECDLP, a private key corresponding to the public key of a user cannot be generated, but validation can be bypassed by replacing the public key alone. The public key replacement attack is mainly possible in existing schemes because the partial key, $D_{A}=\left(R_{A},z_{A}\right)$, generated by the KGC is not related to the public key of the device. In short, it is a CL scheme, and thus lacks a certificate that can authenticate the public key of the signing device. By using this, it is possible to bypass the verification process of the signature, so that the forged signature will be verified by the verifier as if it were proper.

To solve the public key replacement attack, since it is a CL-PKC-based protocol (i.e., without a certificate), the binding between the public key and the identifier must be strengthened. When verifying a public key or verifying a signature signed with a private key, it is only necessary to confirm whether the user has used a public key or a signature made with a key created using a partial key received from the KGC. In summary, the partial public key, $R_{A}$, in the partial key, $D_{A}$, received from device A from the KGC is a tag for verifying the PSK, $z_{A}$, and if the public key, $Z_{A}$, of A created using $z_{A}$ can be confirmed to belong to A, it can be said to be safe against key replacement attacks. In generating $z_{A}$, the value hashed from Equation (4) to $H_{1}$ later serves to verify the public key. If the public key is replaced, it is said that it is safe against public key replacement attacks if the verification of the signature cannot be bypassed using the public key replaced thus. On the other hand, when A’s public key, $PU_{A}$, is replaced by the attacker’s public key, $PU_{A}^{\prime }$, the public key replacement attack is successful if the verifier successfully verifies the forged signature, $\sigma_{A}^{\prime }$, for the message, $m_{A}$.

In the proposed scheme, the form of the individual signature is $\sigma_{A}=\left(\tau_{A},T_{A}\right)$ and the message can be verified normally using the forged public key, $PU_{A}^{\prime }=\left(pu_{A}^{\prime },R_{A}^{\prime },Z_{A}^{\prime }\right)$, associated with the identifier of $A_{I}$. However, it should not be possible to generate the forged signature, $\sigma_{A}^{\prime }=\left(\tau_{A}^{\prime },T_{A}^{\prime }\right)$. The signature generation for $m_{A}$ is according to Equation (8) and, since $A_{I}$ cannot know $t_{A}$ and $PR_{A}$, a valid $\tau_{A}$ cannot be generated. The signature verification is according to Equation (10) and can be generated using Equation (9) and $PU_{A}$. The published information is $T_{A}$, $pu_{A}$, and $R_{A}$ and an attacker who wants to forge it can perform an attack by replacing the public key with $pu_{A}^{\prime }$, $R_{A}^{\prime }$, and $Z_{A}^{\prime }$, and the attacker will try to bypass the verification of $h_{A}$ by generating the same value as $pu_{A}^{\prime }+Z_{A}^{\prime }=h_{A}^{-1}\times P$. However, the verifier can confirm that the public key $Z_{A}^{\prime }$ has not been properly generated using $Z_{A}^{\prime }=R_{A}^{\prime }+H_{1}\left(ID_{A},pu_{A},R_{A}\right)\times P_{pub}$, which can be verified using the public key of the KGC. As for the $\sigma_{AS}=\left(\tau_{AS},T_{AS}\right)$, which is an AAS (aggregate arbitrated signature), it has the same form as the individual signature in Equation (14), so the verifier can correctly verify this even if $A_{I}$ replaces G’s public key. Therefore, $A_{I}$ cannot forge the signature.

#### 5.3.2. Unforgeability from Adversary $A_{\mathrm{II}}$

The adversary $A_{II}$ is a malicious KGC, and since they know *msk*, they have the ability to know all the partial keys of the participating devices. If $A_{II}$ wants to forge A’s signature, they will try to generate one from the partial key, since they lack the ability to replace the public key.

The partial key of A is $D_{A}=\left(R_{A},z_{A}\right)$, where $R_{A}$ is a partial public key and $z_{A}$ is a PSK. The signature generation for the message, $m_{A}$, is via Equation (8) and, since the full private key, $PR_{A}$, generated by the signer, A, is used for this, the KGC cannot forge a signature using only $z_{A}$. It should be impossible to forge the signer’s signature using only external public parameters, including in this $A_{II}$ scenario.

In particular, in the proposed scheme, since the arbitrated signature is performed through a gateway called the arbitrator, it is possible to strengthen non-repudiation. The arbitrated signatures involve this arbitrator, between the signer and the verifier, to protect the validity of the signature and prevent repudiation of the signer; if the gateway performs its arbitrated signature properly, it can prevent forgery of the signature.

## 6. Efficiency Analysis

Another important requirement in the IoT environment is efficiency. In this environment, in which a large number of heterogeneous devices participate in communication, efficiency of the protocol is required so that it can operate even for devices with low computational performance. This includes reducing the amount of computation, and this section compares the existing schemes with the execution time of the proposed CL-AAS.

**Figure 9:**
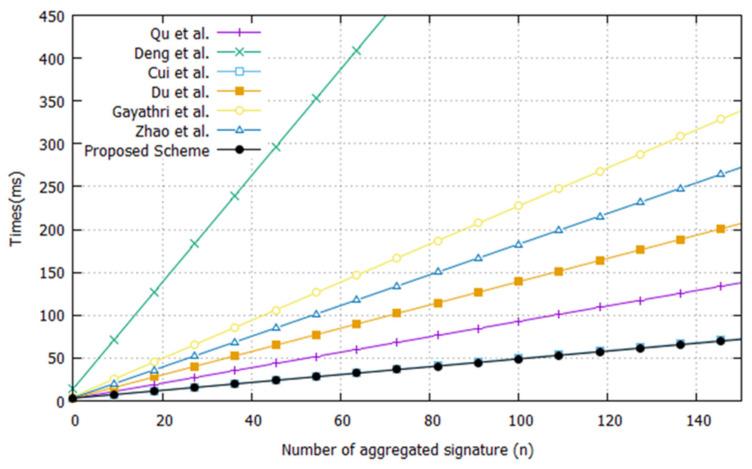
Comparison of execution time between proposed and existing schemes.

The simulation environment constructed in this paper is an Intel i5-4690 processor with 3.50 GHz, 16 GB memory, and Windows 10 operating system. Additionally, to provide security strength like 1024-bit RSA and ECC group, it uses the Koblitz elliptic curve $y^{2}=x^{3}+ax+b\left(mod\;p\right)$, where a=1 and b is a 163-bit random prime defined on $F_{2^{163}}$. Table 2 is a comparison of the execution times with cryptographic operation. The proposed CL-AAS scheme provides computational efficiency compared to the existing [29,30,31,32,33,34] schemes, as shown in Figure 9, by a graph showing the total execution time according to the number of signatures being aggregated, and Table 3. As the number of messages and signatures being aggregated increases, the total times for the aggregated signature and for the verification process increase in direct proportion.

In this proposed scheme, without using a pairing operation, compared with other pairing-free schemes, elliptic-curve cryptography-based addition and multiplication operations are efficiently applied to reduce the total operation time. In addition, since the tag, *T*, for verification is also aggregated for all the messages together, only the part of the public key that the verifier actually acquires and directly calculates is included. Because of this, storage, such as that of a gateway or server, can save space and the verification overhead for the verifier is reduced.

## 7. Conclusions

To maintain the integrity of messages transmitted in an increasingly large IoT service environment, digital signatures for messages are required. Digital signature protocols have been studied for a long time, and many studies are underway to make them suitable for such environments. They are being studied to satisfy various security requirements while respecting the “lightweight” nature of the IoT environment. Although research has been conducted to apply lightweight signature techniques, such as CL-AS, to IoT environments, solutions are needed for the problems of CL-PKC-based schemes, specifically, public key replacement attacks and malicious KGCs. In particular, it is necessary to study solutions that satisfy the requirements for both security and computational efficiency. Therefore, this paper proposes an efficient secure CL-AAS scheme.

The proposed scheme provides the integrity of messages transmitted in an IoT environment using the concepts of an arbitrated signature and an AS (aggregated signature). The role of the AS is to provide efficiency, and that of the arbitrated signature is to enhance non-repudiation by aggregating the arbitrated signatures of a gateway and its devices together. Through this, in this paper, we designed a secure scenario against existing security threats, and considered the security of the gateway, which is an intermediate to transmit data. The proposed scheme is designed to satisfy various security requirements (Section 3), such as such as public key replacement attack, malicious KGC attack, and key leakage. In the existing schemes, as shown in Table 1, there were problems with key leakage and forgery of the message and signature via attacks either by public key replacement or a malicious KGC. To solve this, non-repudiation was strengthened by applying the arbitrated signature of the gateway, and it is possible to provide efficiency by applying an AS to reduce the memory overhead and the verification overhead of the verifier.

In the future, not only as a simple signer and verifier but also in a more complex, grouped, and device-involved environments, the provision of a suitable security scheme is needed. The IoT service may transmit sensitive data, such as personal privacy, depending on the environment. In the future, research on practical security technologies to provide confidentiality and integrity for sensitive data should be conducted.

## Figures and Tables

**Table 1:** Security analysis of various certificateless aggregate signature schemes, including the proposed one.

	Qu et al. [29]	Deng et al. [30]	Cui et al. [31]	Du et al. [32]	Gayathri et al. [33]	Zhao et al. [34]	Proposed Scheme
**Key leakage attack**	**O** Cannot derive key	**X** Can derive key with public parameters	**O** Cannot derive key	**X** Can derive key with public parameters	**O** Cannot derive key	**O** Cannot derive key	**O** Cannot derive key
**Forgery with public key replacement (*A_I_*)**	**X** No identifier binding to public key	**X** No identifier binding to signature	**X** No identifier binding to public key	**O** Binds identifier to public key	**X** No identifier binding to public key	**X** No identifier binding to public key	**O** Binds identifier to public key
**Forgery with KGC master key (*A_II_*)**	**X** Can forge due to public key replacement	**O** Uses two types of signature	**X** Can forge due to public key replacement	**X** Can forge due to key leakage	**O** Uses two types of signature	**O** Sends signature verification tag directly	**O** Uses gateway-arbitrated signature

O (X): scheme is strong (weak) in this category, KGC: key generation center.

**Table 2:** Comparison of execution times with cryptographic operation.

Notations	Description	Run Time (ms)
***T_EM_***	The execution time of scalar multiplication operation in ECC	0.4420
***T_EA_***	The execution time of point addition operation in ECC	0.0018
***T_h_***	The execution time of hash operation	0.0082
***T_E_***	The execution time of scalar exponential operation	5.3100

**Table 3:** Efficiency analysis of the proposed scheme.

	Qu et al. [29]	Deng et al. [30]	Cui et al. [31]	Du et al. [32]	Gayathri et al. [33]	Zhao et al. [34]	Proposed Scheme
**Form of signature**	$\sigma_{i}=\left(U_{i},s_{i}\right)$	$\sigma_{i}=\left(T_{i},B_{i},r_{i},R_{i}\right)$	$\sigma_{i}=\left(R_{i},S_{i}\right)$	$\sigma_{i}=\left(S_{i},v_{i}\right)$	$\sigma_{i}=\left(Y_{1i},u_{i},w_{i}\right)$	$\sigma_{i}=\left(R_{i},\phi_{i}\right)$	$\sigma_{i}=\left(\tau_{i},T_{i}\right)$
**Signing operation**	1H+2EA+2EM	1H+2E+1EA+3EM	H+EA+2EM	2H+2EA+3EM	3H+3EA+5EM	2H+2EA+2EM	1H+2EA+2EM
**Verifying operation**	2EA+3EM	E+1EA+4EM	2H+2EA+3EM	3H+3EA+3EM	2H+3EA+5EM	2H+3EA+4EM	1H+2EA+2EM
**Aggregating operation**	nEA	2nEA	nEA	nEA	$3n\left(EA+EM\right)$	nEA	$1H+\left(2n+3\right)EA+2EM$
**Aggregated verifying operation**	$n\left(1H+4EA+2EM\right)+1EA+1EM$	$n\left(1H+2EA+2EM+E\right)+1EM$	$n\left(2H+2EA+1EM\right)+2EM+2EA$	$n\left(3H+4EA+3EM\right)+2EA+1EM$	$n\left(1H+1EA+2EM\right)+2EA+1EM$	$n\left(2H+4EA+4EM\right)+3EA+2EM$	$n\left(1H+2EA+1EM\right)+1H+3EA+1EM$
**Total operations**	$\left(n+1\right)H+\left(5n+4\right)EA+\left(2n+6\right)EM$	$\left(n+1\right)H+\left(n+2\right)E+\left(4n+2\right)EA+\left(2n+8\right)EM$	$\left(2n+3\right)H+\left(3n+5\right)EA+\left(n+7\right)EM$	$\left(3n+5\right)H+\left(5n+7\right)EA+\left(3n+7\right)EM$	$\left(n+5\right)H+\left(4n+8\right)EA+\left(5n+11\right)EM$	$\left(2n+4\right)H+\left(5n+8\right)EA+\left(4n+8\right)EM$	$\left(n+3\right)H+\left(4n+10\right)EA+\left(n+7\right)EM$
**Total operation time (ms, n = 100)**	92.7874	635.1078	49.5076	139.1076	227.4574	182.9232	48.8766

H: One-way hash function, E: Modular exponential operation. EA: Elliptic curve addition operation, EM: Elliptic curve scalar multiple operation. See references for definitions of variables in the forms of the signatures.
